# Racial disparities in access to DBS: results of a real-world U.S. claims data analysis

**DOI:** 10.3389/fneur.2023.1233684

**Published:** 2023-08-01

**Authors:** Michael Frassica, Drew S. Kern, Mitra Afshari, Allison T. Connolly, Chengyuan Wu, Nathan Rowland, Juan Ramirez-Castaneda, Mwiza Ushe, Claudia Salazar, Xenos Mason

**Affiliations:** ^1^Abbott Laboratories, Austin, TX, United States; ^2^Department of Neurology, University of Colorado School of Medicine, Aurora, CO, United States; ^3^Department of Neurosurgery, University of Colorado School of Medicine, Aurora, CO, United States; ^4^Department of Neurological Sciences, Rush University Medical Center, Chicago, IL, United States; ^5^Department of Neurological Surgery, Vickie and Jack Farber Institute for Neuroscience, Thomas Jefferson University, Philadelphia, PA, United States; ^6^Department of Neurosurgery, Medical University of South Carolina, Charleston, SC, United States; ^7^Methodist Physicians, Neurosurgery and Neurology Specialists, San Antonio, TX, United States; ^8^Department of Neurology, Washington University, St. Louis, MO, United States; ^9^Department of Neuroscience, Medical University of South Carolina, Charleston, SC, United States; ^10^Department of Neurology, University of Southern California Keck School of Medicine, Los Angeles, CA, United States; ^11^Department of Neurological Surgery, University of Southern California Keck School of Medicine, Los Angeles, CA, United States

**Keywords:** deep brain stimulation, Parkinson’s disease, essential tremor, medicare, race, ethnicity, telehealth, movement disorder

## Abstract

**Introduction:**

Deep brain stimulation (DBS) is an effective and standard-of-care therapy for Parkinson’s Disease and other movement disorders when symptoms are inadequately controlled with conventional medications. It requires expert care for patient selection, surgical targeting, and therapy titration. Despite the known benefits, racial/ethnic disparities in access have been reported. Technological advancements with smartphone-enabled devices may influence racial disparities. Real-world evidence investigations can shed further light on barriers to access and demographic disparities for DBS patients.

**Methods:**

A retrospective cross-sectional study was performed using Medicare claims linked with manufacturer patient data tracking to analyze 3,869 patients who received DBS. Patients were divided into two categories: traditional omnidirectional DBS systems with dedicated proprietary controllers (“traditional”; *n* = 3,256) and directional DBS systems with smart controllers (“smartphone-enabled”; *n* = 613). Demographics including age, sex, and self-identified race/ethnicity were compared. Categorical demographics, including race/ethnicity and distance from implanting facility, were analyzed for the entire population.

**Results:**

A significant disparity in DBS utilization was evident. White individuals comprised 91.4 and 89.9% of traditional and smartphone-enabled DBS groups, respectively. Non-White patients were significantly more likely to live closer to implanting facilities compared with White patients.

**Conclusion:**

There is great racial disparity in utilization of DBS therapy. Smartphone-enabled systems did not significantly impact racial disparities in receiving DBS. Minoritized patients were more likely to live closer to their implanting facility than White patients. Further research is warranted to identify barriers to access for minoritized patients to receive DBS. Technological advancements should consider the racial discrepancy of DBS utilization in future developments.

## 1. Introduction

Deep brain stimulation (DBS) is an established therapy for the motor symptoms of many movement disorders (1). This therapy is standard-of-care for medication-refractory Parkinson’s disease (PD), Essential Tremor (ET), and Dystonia (DYT) but incurs high healthcare costs and often requires long-term access to an expert, multidisciplinary team to implant the device, manage device programming and surgical complications, and down titrate medications (1). Prior studies have demonstrated disparities in access to PD-specialized care among underrepresented groups in medicine, and similarly to DBS (2–7). Such underrepresented groups will be referred to as ‘minoritized’ herein, recognizing the systemic social element of minority race/ethnicities (8). In a 2014 analysis of DBS utilization in approximately 690,000 Medicare beneficiaries with PD, significant disparities were associated with race and neighborhood socioeconomic status (9). In a 2022 analysis of Nationwide Inpatient Sample (NIS) data from 2002 to 2018 that documented 50,837 patients receiving DBS patients, DBS utilization had risen 82% over 16 years but racial disparities remained consistent. Comparing 2002–2009 and 2010–2018 data, White patients were five times more likely to undergo DBS than Black patients in the US (6).

Much focus has been placed on increasing access to DBS through telemedicine and remote-care platforms over the last 5 years (10–12). Newer-generation smartphone-controlled DBS systems can allow for remote programming that traditional DBS systems with proprietary controllers cannot. However, it is unclear if these modern systems introduce further disparity compared to traditional DBS systems due to internet access. In a 2021 analysis of internet usage amongst Americans, while there was a 9% difference in home broadband use between White and Black households, there was only a 2% difference in smartphone use (13). Smartphone-enabled, telemedicine-compatible DBS systems may have the potential to provide improved access to specialized care for traditionally underserved populations. The effect of smartphone-enabled DBS systems on racial disparity in DBS has not been previously reported.

Herein, we analyze a large dataset of US Medicare beneficiary data from patients who have received smartphone-enabled versus traditional DBS systems. Medicare is federal health insurance for people who are 65 years or older or who have disabilities or end-stage renal disease. Smartphone-enabled DBS systems herein are defined as systems with directional lead capability that are controlled using mobile operating system (OS) devices (i.e., smart devices/tablets) communicating via Bluetooth, which were only manufactured by St. Jude Medical/Abbott in the window of this analysis. “Traditional” DBS systems during this timeframe offered omnidirectional stimulation and used radio-frequency controllers that require dedicated devices from the DBS system manufacturer. Using these comparator cohorts, we aimed to characterize and compare the age, sex, and race/ethnicity of Medicare beneficiaries receiving these devices. In addition, we investigated the effect of race/ethnicity on distance between the beneficiary’s home and the location of medical center where the DBS system was implanted.

## 2. Methods

The present study was an observational, non-randomized, contemporaneous-cohort, retrospective study of Medicare claims linked with manufacturer patient device tracking (PDT) data. Patients were analyzed and categorized through correlation of Medicare claims data and manufacturer patient data tracking (PDT) as previously described in Wu et al. (14). Center for Medicare and Medicaid Services (CMS) insurance claims data contains information on healthcare system utilization, including content and date of medical procedures/diagnoses, filled prescriptions, and death. The study herein utilized device data from Abbott, which maintains a PDT database with device type, implant date, and basic patient demographic information, to match patients within the CMS claims data.

### 2.1. Patient population

Patients with DBS systems implanted between October 6, 2016 and December 31, 2018 in the US were eligible for this analysis. The smartphone-enabled DBS group exclusively included Abbott Infinity DBS Systems, as it was the only available system with such capabilities in the identified time window. The “traditional” DBS system group is assumed to be majority Medtronic DBS systems, as this was the only other system available until the Boston Scientific Vercise system was approved in the US in 2018. Prior to 2019, both the Medtronic and Boston Scientific systems utilized radiofrequency-communication proprietary DBS controllers, while the Abbott system was smartphone-enabled with iOS^®^ devices (Apple Inc., Cupertino, CA). While more modern DBS systems are available across manufacturers now, this unique time window provides an opportunity to compare system types.

Inclusion criteria included enrollment in Fee For Service (FFS) Medicare Part A and B, age > 18, primary diagnosis of PD or ET (excluding implants for other disorders), implanted with a DBS lead, and implanted with an implantable pulse generator (IPG) within 3 months of lead implantation. Patients had to have the total system including lead, connection cables and IPG implanted for a minimum of 1 month. Medicare enrollment data one year prior to and 3 months after lead implant was required. To isolate patients undergoing first-time implantation of a DBS system, patients were excluded if they had history of DBS-related procedures within the required time window of Medicare enrollment data. To ensure the same data was available for all patients, those enrolled in Medicare HMO or Medicare Advantage, wherein coverage is provided by private insurance companies approved by Medicare, were excluded. Specific procedure and diagnosis codes used for patient population determination can be found in Wu et al. (14).

### 2.2. Data analysis

Patients were allocated to DBS system groups by linking Medicare patients to Abbott PDT through probabilistic linking methods (14, 15). Patients matching uniquely at the first iteration—with exact matches of all indirect identifiers available—were assigned to the “smartphone-enabled” DBS group. Patients with non-unique/duplicate matches or matches utilizing default values or other variants for missing/incomplete indirect identifiers (“near-matches”) were removed from further analysis. All remaining unmatched patients were assigned to the “traditional” DBS group.

Patient age, sex, and race/ethnicity were determined from the CMS Master Beneficiary Summary File (MBSF) at the time of DBS lead implant. Self-identified race, originating from the Medicare enrollment database, was collected as identified by patients with outputs of Asian, Black, Hispanic, North American Native, White, Other, or Unknown. Further racial delineations and option for ‘multiracial’ were not available in the database. Due to low number of subjects identifying as a race other than white, analyses were conducted between two groups: White and minoritized. In the minoritized group, Asian, Black, Hispanic and North American Native beneficiaries were pooled, and beneficiaries of unknown or other race/ethnicity were excluded from the analysis. Patient home location was determined from the beneficiary zip code, and location of the DBS lead implant clinic was determined from the zip code of the facility at which the procedure was performed using the NPI registry. The distance from beneficiary to lead implant was determined using the SAS function *zipcitydistance*.

Patient demographics are reported as mean ± standard deviation for continuous variables and counts with percentages for categorical variables. Mean age difference between groups was compared using a *t*-test. Chi-squared tests were used to detect differences in demographics between system groups and race/distance groups. Comparison of distance to implanting center for White vs. minoritized patients was done using a Wilcoxon rank sum test. The threshold for statistical significance was *p* < 0.05 without correction for multiple comparisons. In compliance with CMS data privacy policies, values including 10 or fewer patients are treated as *n* < 11.

## 3. Results

Initial analysis of Medicare patients found 5,998 patients received first-time DBS lead implants between October 6, 2016 and December 31, 2018. This study included 3,869 patients who met the inclusion and exclusion criteria and probabilistic matching requirements. Of those patients, 613 (15.8%) received smartphone-enabled DBS systems and 3,256 patients (84.2%) received traditional. Based on unique organization NPI (rather than unique facility or hospital organization), implants occurred at 282 US sites.

### 3.1. Demographics

As seen in Table 1A, patients in both the smartphone-enabled and traditional DBS groups had similar characteristics. The average age was 70.9 years and females made up approximately 38% of the study population. Age and sex of patients was not significantly different between groups (Age (*t*-test): *p* = 0.950; Sex (chi-squared test): *p* = 0.990). Patients with traditional and smartphone-enabled DBS systems were predominantly White (91.4 and 89.9%, respectively), which greatly exceeded the proportion of the overall Medicare population who is White (75%–76%; Table 1B) (16). Black individuals comprise 10% of the overall Medicare population, but 2% or less of the patients receiving DBS implants in this cohort. Hispanic individuals comprise 9% of Medicare beneficiaries, but less than 2% of either DBS system group. There was a trend toward greater usage of smartphone-enabled DBS Systems among minoritized patients, but this was not statistically significant (chi-squared test: *p* = 0.181). Black individuals comprised a higher proportion of the total smartphone-enabled implants compared to traditional implants, 1.1% & 2.4% for traditional and smartphone-enabled DBS groups, respectively, although not statistically significant.

**Table 1:** Patient demographics for traditional and smartphone-enabled DBS systems.

1A	Demographic	Traditional DBS System (*N* = 3,256)	Smartphone-enabled DBS System (*N* = 613)
	Age, years	70.9 ± 6.9	70.9 ± 7.1
	Sex, female	1,242 (38.1%)	234 (38.2%)
	Race/Ethnicity		
	White	2,977 (91.4%)	551 (89.9%)
	Black	36 (1.1%)	15 (2.4%)
	Asian	35 (1.1%)	<11 (<1.8%)
	Hispanic	46 (1.4%)	<11 (<1.8%)
	North American Native	<11 (<0.3%)	<11 (<1.8%)
	Other	47 (1.4%)	11 (1.8%)
	Unknown	106 (3.3%)	19 (3.1%)

1B	Race/Ethnicity Distribution in Medicare Beneficiaries	2016	2017	2018	2019
	White	76%	75%	75%	75%
	Black	10%	10%	10%	10%
	Hispanic	9%	9%	9%	9%
	Asian/Pacific Islander	4%	4%	4%	4%
	American Indian/Alaska Native	1%	<1%	<1%	1%
	Multiple	1%	1%	1%	1%

(A) Patient demographics were compared for patients receiving traditional DBS systems versus smartphone-enabled DBS systems. (B) Race distribution of all Medicare beneficiaries per year from 2016–2019 provided for comparison from https://www.kff.org/medicare/state-indicator/medicare-beneficiaries-by-raceethnicity/ (16).

### 3.2. Distance

**Figure 1:**
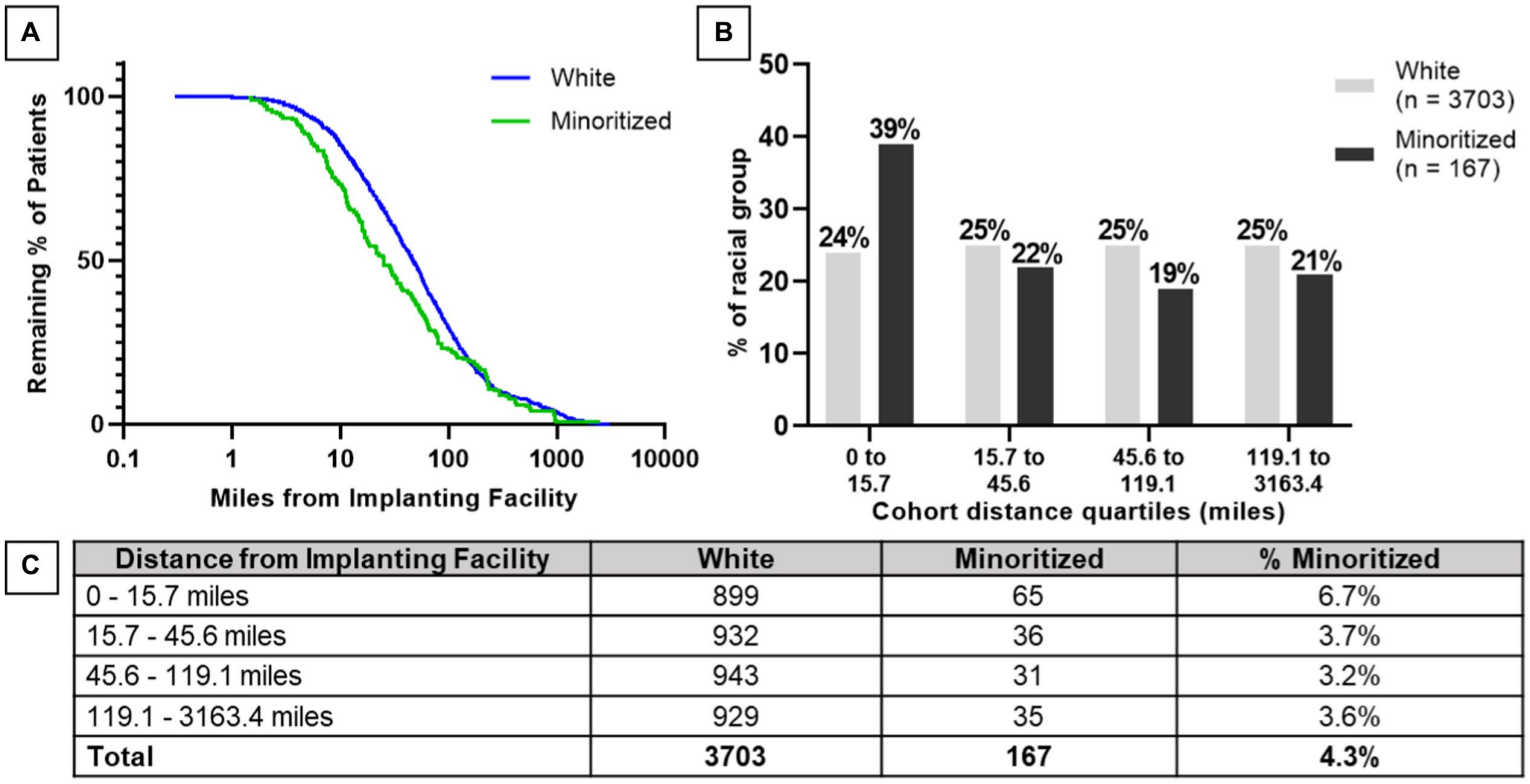
Population-level comparison of distance from DBS implant center to patient’s home among White and minoritized groups. **(A)** Survival comparison of minoritized and White patients based on distance from implanting facility presented on a logarithmic scale (log-rank test: *p* = 0.0103). **(B)** Distribution of patient groups in the four quartiles of the full cohort are presented in bar format. Minoritized patients were significantly more likely to be in the first quartile group compared to White patients (chi-squared test: *p* < 0.001). **(C)** Patient counts within each quartile of distance from the DBS implant center for minoritized and White patients.

A secondary analysis was performed to examine the distance between the DBS patient’s home and the clinic at which they received their DBS lead implant. For this analysis, Asian, Black, Hispanic, and North American Native beneficiaries were grouped into a minoritized category due to small sample size among racial/ethnic subgroups. When mean distance for White patients (*N* = 3,709), 147 ± 313 miles, was compared to that of minoritized patients (*N* = 168), 117 ± 266 miles, a statistically significant difference was detected between the two groups (Wilcoxon rank sum test; normal approximation one-sided: *p* < 0.0001) where minoritized patients lived closer to the implanting clinic. Figure 1A graphically represents the two demographic groups in a survival analysis format on a logarithmic scale, where drop-offs in the two curves occur as beneficiaries live at that distance. These curves also demonstrate a significant difference in the two populations (log-rank test: *p* = 0.0103). The two groups were then divided into distance ranges based on quartiles of the entire population’s distances from implanting facility. Minoritized DBS patients were significantly more likely to be in the first quartile range (shortest distance from home to implanting center) compared to White DBS patients (chi-squared test: *p* < 0.001) (Figure 1B). In all distance ranges shown in Figure 1C, self-reported minoritized beneficiaries were underrepresented compared to the minoritized representation of 23.5–24.0% expected from Medicare beneficiaries (Table 1).

## 4. Discussion

Our results indicate that there remains a significant racial disparity in patients undergoing DBS. Approximately 90% of the population receiving DBS implantation self-identified as White, which is notably higher than Medicare beneficiaries (75%–76%) (Table 1), and is insufficiently explained by known ethnic differences in PD and ET prevalence (16). As such, minoritized populations are under-represented in receipt of DBS therapy, including notably Black individuals for whom sample size allows for an accurate comparison of proportion. Finding explanations for this under-representation remains a critical area for future investigation, including but not limited to biological (genetic and environmental contributors to prevalence and expression of disease), psycho-social (individual and community behaviors relating to interactions with medical systems), and structural (geographic, political and economic factors) (4, 6, 9). Determining a specific explanation for the source of the disparities that we identify is outside the scope our of analysis; indeed multiple explanations may simultaneously contribute, and further research in DBS health disparity and health equity is needed. There was minimal difference in distribution of race/ethnicity between the smartphone-enabled and traditional DBS groups, which may suggest that no additional technological access barriers exist with the smartphone-enabled system. In fact, the proportion of smartphone-enabled DBS systems received by Black patients was more than twice the proportion of traditional systems, although this was not statistically significant with the small sample size. Further investigation of whether non-white patients may favor smartphone-enabled DBS systems is warranted.

Disparity was seen in use of DBS based on distance between patient’s implanting facility and home. Minoritized patients implanted with DBS were significantly more likely to be located closer to their implanting facility. Furthermore, when split by distance quartiles, the distribution of minoritized individuals, as compared to White individuals, favored the closest quartile to the implanting facility. If this reflects the true distribution of DBS-eligible patients, this geography can be leveraged to provide non-White/minoritized patients easier access to DBS care. If instead this represents a true disparity in access to DBS, many factors may be responsible including referral patterns or socioeconomics (2, 9, 17, 18). Our study highlights that identifying contributing factors to disparities is an important area of future research.

One potential advantage of smartphone-enabled DBS is potential to perform telehealth, which may improve patient access (11, 19). High feasibility for telehealth PD visits has been supported in randomized and non-randomized studies with high patient and clinician satisfaction (20, 21). A recent analysis of a single movement disorder practice showed that during the COVID-19 pandemic there was no disparity in sex and race for conversion to telemedicine (22). In a recent survey conducted by the Parkinson’s Foundation, over a third of patients with PD indicated difficulty traveling to their clinic. In the same population, those who completed telehealth visits with remote programming showed satisfaction with replacement of an in-person visit (23). It remains unclear the impact of technological advances in DBS such as smartphone-based telehealth capability on racial disparity in use of DBS.

Limitations exist in this analysis. Only patients entirely on Medicare were included, excluding private insurance and Medicare Advantage (Part C)/Medicare HMO. Patients younger than 65 years of age were unlikely to have been included in the analysis unless enrolled in Medicare for disability. The majority of patients receiving DBS earlier than 65 or covered by other insurances are unable to be analyzed by this method. It is unclear if this population may experience different trends in care. Second, some patients were removed from further analysis due to probabilistic matching methods, which may asymmetrically impact the trends identified herein. A selection/exclusion bias (i.e., minoritized-patients living further from the implanting center being selectively excluded from our analysis) is unlikely to have occurred by our methods, given the liberal inclusion criteria. Information not covered herein includes socioeconomic demographics, referral pathway to implant center, disease and diagnostic differences (PD vs. ET) and regional differences in the adoption rate of new devices, which could relate to practitioner or industry factors related to commercialization and dissemination of these devices. These factors are important to consider in future research but outside the analytical scope of our analyses. The external validity of our analysis is not limited to a single disease, but instead applies across disease states; this may be considered both an advantage, and a weakness of this study depending on the goals of our readers. Aggregate Medicare data does not include information on disease characteristics; while relevant to a discussion of DBS disparities, single-center data would be a necessary complement to aggregate data in further research. The limited number of patients of each self-identified race/ethnicity necessitated aggregation of subgroups into a broad categorization of “minoritized,” and disparities may exist between races/ethnicities that we were not able to assess individually. Furthermore, people of some races/ethnicities may self-identify as White, impacting this population. Finally, telehealth-capability was not yet available for smartphone-enabled DBS systems at the time of this analysis, which may have further impact on these trends.

This analysis of ‘real-world’ manufacturer and Medicare claims provides a broad representation of care trends in DBS. Specifically, it demonstrates that non-White races/ethnicities may be underserved in DBS therapy. Contributing factors to these results on a regional or individual practice-level warrant further investigation to identify opportunities for enhanced access for patients. The use of smartphone programmer technology does not appear to influence racial disparity in DBS. The distance from a DBS center may have a disproportionate influence on provision of DBS therapy to racial minoritized groups. Future research is needed to determine if telehealth capability with smartphone programmers will enhance access to standard-of-care DBS therapy for underserved populations.

## Data availability statement

Publicly available datasets were analyzed in this study. Centers for Medicare and Medicaid Services data can be requested under an approved research protocol via ResDAC (www.resdac.org). Restrictions apply to the availability of data generated and analyzed during this study to preserve patient confidentiality and because data are used under license. Requests to access these datasets should be directed to ResDAC, resdac@umn.edu.

## Ethics statement

The studies involving human participants were reviewed and approved by Western IRB. Written informed consent for participation was not required for this study in accordance with the national legislation and the institutional requirements.

## Author contributions

DK, MA, CW, NR, JR-C, MU, and CS: review and critique of manuscript. MF, AC, and XM: manuscript content, preparation, and analysis. All authors contributed to the article and approved the submitted version.

## Funding

Abbott provided funding for this study.

## Conflict of interest

MF and AC are employees at Abbott Laboratories. DK has served as an advisor for Colorado Clinical and Translational Sciences Institute (CCTSI) Data Safety Monitoring Board, and Medical Boards for Boston Scientific, Medtronic, and AbbVie Pharmaceutics; received honorarium from AbbVie Pharmaceutics and Boston Scientific; received grants from the Boston Scientific, Medtronic, University of Colorado Department of Neurology, and the Parkinson’s Foundation. CW is a consultant for Abbott, Medtronic, and Boston Scientific. MU has research sponsored by the NIH and participates in clinical trials with Biogen (SCA3) and AbbVie (subcutaneous levodopa infusion). JR-C is part of consulting/speaker bureaus for: Deep brain stimulation: Abbott and Medtronic; carbidopa/levodopa enteric system: Duopa (Abbvie); carbidopa levodopa extended-release capsules: Rytary (Amneal); inhaled levodopa: Inbrija (Acorda); injectable apomorphine: Apokyn, rimabotulinum toxinB: Myobloc, safinamide: Xadago (Supernus). MA receives research grant support from the Parkinson Study Group, is the site-PI and thus receives salary support for the SPARX3 clinical trial (Funded by NIH/NINDS, 1U01NS113851-01) and receives consulting fees from Abbott. XM is a consultant for Abbott. The authors declare that this study received funding from Abbott Laboratories. Abbott Laboratories funded the study design, data collection, and analysis, and employees of Abbott Laboratories (MF and AC) contributed to all phases of manuscript development.

The remaining authors declare that the research was conducted in the absence of any commercial or financial relationships that could be construed as a potential conflict of interest.

The handling editor MS declared a shared affiliation, though no other collaboration, with one of the authors JR-C at the time of the review.

## Publisher’s note

All claims expressed in this article are solely those of the authors and do not necessarily represent those of their affiliated organizations, or those of the publisher, the editors and the reviewers. Any product that may be evaluated in this article, or claim that may be made by its manufacturer, is not guaranteed or endorsed by the publisher.
